# Tunable integration of absorption-membrane-adsorption for efficiently separating low boiling gas mixtures near normal temperature

**DOI:** 10.1038/srep21114

**Published:** 2016-02-19

**Authors:** Huang Liu, Yong Pan, Bei Liu, Changyu Sun, Ping Guo, Xueteng Gao, Lanying Yang, Qinglan Ma, Guangjin Chen

**Affiliations:** 1 State Key Laboratory of Heavy Oil Processing, China University of Petroleum, Beijing 102249, P. R. China; 2 State Key Laboratory of Oil and Gas Reservoir Geology and Exploitation, Southwest Petroleum University, Chengdu 610500, China

## Abstract

Separation of low boiling gas mixtures is widely concerned in process industries. Now their separations heavily rely upon energy-intensive cryogenic processes. Here, we report a pseudo-absorption process for separating low boiling gas mixtures near normal temperature. In this process, absorption-membrane-adsorption is integrated by suspending suitable porous ZIF material in suitable solvent and forming selectively permeable liquid membrane around ZIF particles. Green solvents like water and glycol were used to form ZIF-8 slurry and tune the permeability of liquid membrane surrounding ZIF-8 particles. We found glycol molecules form tighter membrane while water molecules form looser membrane because of the hydrophobicity of ZIF-8. When using mixing solvents composed of glycol and water, the permeability of liquid membrane becomes tunable. It is shown that ZIF-8/water slurry always manifests remarkable higher separation selectivity than solid ZIF-8 and it could be tuned to further enhance the capture of light hydrocarbons by adding suitable quantity of glycol to water. Because of its lower viscosity and higher sorption/desorption rate, tunable ZIF-8/water-glycol slurry could be readily used as liquid absorbent to separate different kinds of low boiling gas mixtures by applying a multistage separation process in one traditional absorption tower, especially for the capture of light hydrocarbons.

Separation of low boiling gas mixtures is widely involved in process industries, especially in petrochemical industry. Now their separations heavily rely upon energy-intensive distillation-based cryogenic technologies, which present a class of the most important and also the most costly processes in the chemical industry^1^,^2^. Separation of pyrolysis gas in the production of ethylene (C_2_H_4_) is a typical example. The operation temperature of cool tank for separating hydrogen (H_2_) with methane (CH_4_) is as low as 113 K and the separation temperature for CH_4_ with C_2_H_4_ also reaches 163 K. Motivated by the staggering energy costs associated with these deep cool separations, developing the substitute technologies has become a very active area^3^,^4^,^5^,^6^,^7^. Membrane and adsorption are two main substitute approaches through which low boiling gas mixture can be separated under normal temperature.

As an emerging class of porous materials, metal-organic frameworks (MOFs)^8^ are regarded as promising candidates for gas adsorption separations. MOFs are three-dimensional networks of metal clusters that are connected with organic linkers. By changing the metal and/or linker we can synthesize millions of different materials. Moreover, one can use this tunability to synthesize a material that has exactly the right pore volume, surface area, and selectivity to efficiently realize gas manipulations^9^,^10^,^11^,^12^,^13^,^14^. In contrast to the enormous attention given to the H_2_ adsorption/storage and carbon dioxide (CO_2_) capture in the past decades^14^,^15^,^16^,^17^,^18^,^19^, currently, there are more and more reports on the adsorption-based separation of gas/hydrocarbon and light hydrocarbons using MOFs as adsorbents^20^,^21^,^22^,^23^,^24^,^25^,^26^,^27^,^28^,^29^,^30^,^31^.

The enthusiasm of the scientific community about MOFs and other nano-porous materials as solid-adsorbents, however, does not yet resonate in the process engineering community^2^,^32^,^33^. The fact is due to two significant advantages of the use of cryogenic separation technology. The most remarkable advantage of distillation is multistage separations can be realized in a single equipment and very high purities of products could be achieved. In these points, pressure-swing adsorption (PSA) is uncompetitive. Another advantage of distillation approach is only fluids are involved in the process, thus heat integration is more convenient. Perfect heat integration can also conserve energy efficiently.

Membrane process is another approach for the separation of low boiling gas mixtures at normal temperature^31^,^34^,^35^. MOF materials have been applied to the synthesis of membrane in recent years^36^,^37^,^38^,^39^,^40^. The main bottlenecks for the application of membrane are the high cost of membrane materials, and usually the separation performances of them have come up to short when characterized as thin membranes with gas mixtures at industry relevant operating conditions^41^. Multistage separation for membrane separation is also difficult to be realized.

In order to take the advantages of both MOFs and distillation, we recently proposed a so called absorption-adsorption hybrid method^42^. In this method, carefully chosen MOFs were dispersed in liquid to form stable and flowable slurry and then used for gas separation just like liquid absorption. We found ZIF-8 could be dispersed in glycol evenly and its adsorption ability for CO_2_ remained, while that for other gas species (H_2_, N_2_, CH_4_, *et al*.) decreased dramatically. Thus the ZIF-8/glycol slurry could be used for CO_2_ capture with high selectivity^42^.

In this work we demonstrate that the key factor affecting the separation selectivity of absorption-adsorption hybrid method is the penetration selectivity of liquid membrane formed by solvent molecules around the ZIF-8 particles for different gas species. Green solvents like water and glycol were used to adjust the penetration selectivity of liquid membrane aiming at enhancing the separation selectivity of ZIF-8 slurry for different kinds of low boiling gas mixtures. By this way, ZIF-8/water-glycol slurry could be tuned to integrate absorption, membrane, and adsorption into a pseudo-absorption process with enhanced separation efficiency over single absorption, membrane or adsorption process and easily applied in traditional multistage separation process like distillation for separating low boiling gas mixtures near normal temperature.

## Results and Discussion

### Tunable Liquid Membrane

**Figure 1:**
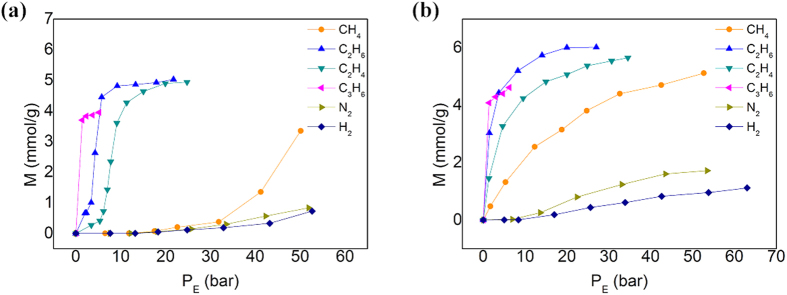
Adsorption isotherms of different gas species CH_4_, C_2_H_6_, C_2_H_4_, C_3_H_6_, N_2_, and H_2_on ZIF-8. (**a**) On ZIF-8 suspended in glycol with a mass fraction of 18.3%. (**b**) On ZIF-8 suspended in water with a mass fraction of 16.7% at 293.15 K.

**Figure 2:**
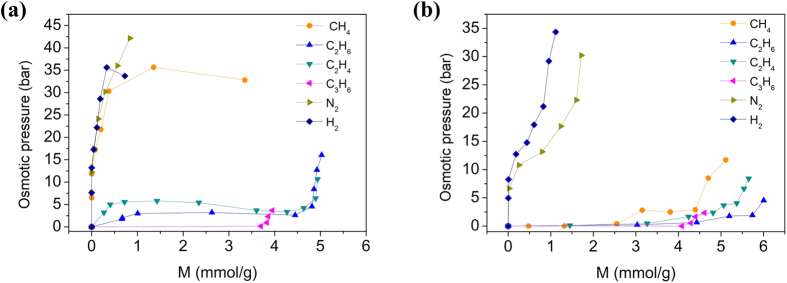
The variation of osmotic pressure of gas species with the gas uptakes in ZIF-8 framework. (**a**) For ZIF-8/glycol slurry with a ZIF-8 mass fraction of 0.183. (**b**) For ZIF-8/water slurry with a ZIF-8 mass fraction of 0.167 at 293.15 K.

We first measured the sorption isotherms of a series of low boiling gas species on ZIF-8 slurry formed by mixing ZIF-8 with glycol or water. We find the shapes of sorption isotherms are strongly solvent type dependent. Figure 1a,b show the adsorption isotherms of six different gas species (CH_4_, C_2_H_6_, C_2_H_4_, C_3_H_6_, N_2_, and H_2_) in ZIF-8 suspended in glycol and water, respectively. By comparing them with the isotherms on pure solid ZIF-8 (Supplementary Fig. S1), we see that most gas species become more difficult to enter the pores of ZIF-8 suspended in glycol. In other words, it requires excess pressures for these gas species to achieve the same uptakes in the suspended ZIF-8 as those in pure solid ZIF-8. This kind of excess pressure just likes the osmotic pressure of a semi-permeable membrane^38^. This phenomenon demonstrates that glycol molecules absorbed on the outer surface of ZIF-8 particles work as a liquid membrane. The formation of liquid films on the solid surfaces have been widely studied^43^,^44^. However, most involved solid surfaces are not porous. Whether glycol or water could form liquid membranes surrounding porous ZIF-8 particles as well as their structures reserve further investigations. Here we put our emphasis on the application of the excess pressure for gas separation. In order to be consistent with traditional membrane processes, here we still call the excess pressure as *osmotic pressure* and define it as,


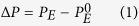


where 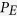 is the vapor-slurry equilibrium pressure at a given gas uptake in ZIF-8 and 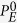 is the vapor-solid equilibrium pressure at the same gas uptake in ZIF-8. The calculated osmotic pressures of different gas species on ZIF-8/glycol slurry and ZIF-8/water slurry were given in Fig. 2a,b, respectively.

Obviously, there are big differences in osmotic pressures between lighter and heavier components for both ZIF-8/glycol slurry and ZIF-8/water slurry. There is no observable osmotic pressure for propene (C_3_H_6_). Lighter molecules such as N_2_ and H_2_ have very high osmotic pressures. As shown by Fig. 2a,b, when the solvent is changed from glycol to water, the osmotic pressure disappears for most heavier species, C_3_H_6_, C_2_H_6_, C_2_H_4_ and CH_4_, in lower gas uptake range, while there are still high osmotic pressures for lighter species N_2_ and H_2_. This phenomenon implies that ZIF-8/water slurry could be used to separate light hydrocarbons from N_2_ and H_2_ promisingly.

**Figure 3:**
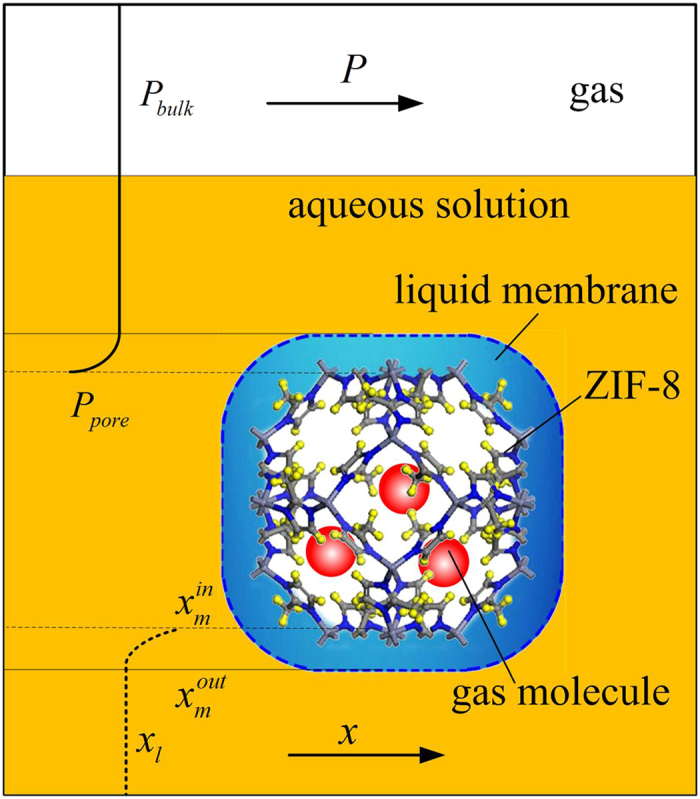
Schematic diagram of absorption-membrane-adsorption process of gas species in ZIF-8 slurry. *P*_bulk_ is the pressure of bulk gas phase and liquid phase, *P*_pore_ is the pressure inside the pores of ZIF-8, 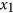 is the mole fraction of one gas species in bulk liquid phase, 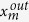 and 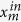 are the mole fractions of the gas species at the outer boundary and the inner boundary of the liquid membrane.

Figure 3 shows the possible mechanisms for absorption-membrane-adsorption process for a gas species in ZIF-8 slurry and the origin of osmotic pressure. Although gas molecules can dissolve in both solvent and ZIF-8, the gas uptake in the slurry was dominated by the adsorption of ZIF-8. Large difference between the concentration of gas molecules in solvent phase and solid phase may result in a reverse gas concentration gradient across the liquid membrane, i.e., the gas concentration at the inner boundary might be larger than that at the outer boundary of the membrane. Without a positive excess pressure, the gas molecules could not transfer through the liquid membrane from the outside to the inside. That might be why excess or osmotic pressure originates. Obviously, larger reverse gas concentration gradient corresponds to higher osmotic pressure. Because larger gas molecules have bigger solubility in glycol or water (see Supplementary Table S1), their reverse concentration gradients within the liquid membrane are smaller. Hence heavier gas species have lower osmotic pressures. The gas solubility in solvent could be increased by decreasing temperature. We do find osmotic pressure decreases with decreasing temperature as shown by Supplementary Fig. S2.

As shown in Fig. 2, osmotic pressure increases rapidly with increasing gas uptake within ZIF-8 even for heavier gas species in higher gas uptake range. This is because the solubility of gas molecules in solvent cannot increase with a same speed as gas uptake within ZIF-8, resulting in the reverse gas concentration gradient in the liquid membrane increasing rapidly with increasing gas uptake.

The osmotic pressure depends not only on gas solubility in solvent, but also the type of solvent itself. All gas species have lower solubilities in water than in glycol. However, they have smaller osmotic pressures in ZIF-8/water slurry than in ZIF-8/glycol slurry. The liquid membrane should be of some particular structure dominated by the interactions among solvent molecules and the function groups or atoms located on the surface of solid particles. Glycol molecules are more ZIF-philic than water molecules because of their -CH group. Thus glycol molecules are easier to be adsorbed on the surface of ZIF-8 particles and self-assemble a tighter membrane. Most gas molecules are difficult to penetrate them. Zhang *et al*.^45^ also found the adsorption amount of alcohols on ZIF-8 is much higher than that of water due to the strong hydrophobicity of ZIF-8.

**Figure 4:**
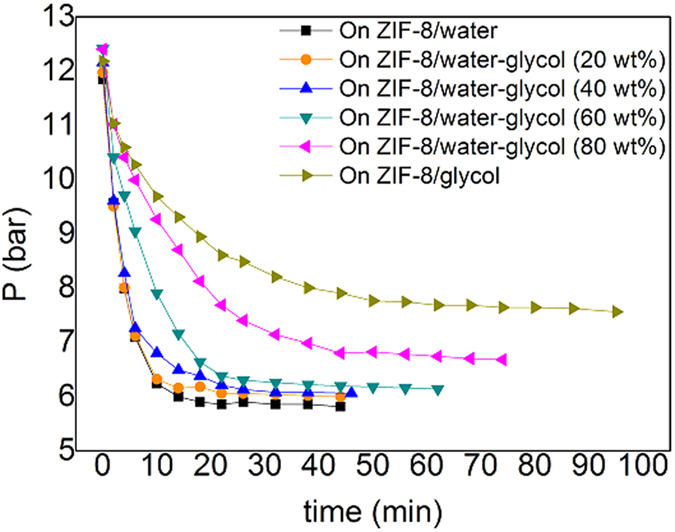
Ab(d)orption kinetic profiles of C_2_H_4_ on ZIF-8/water-glycol slurry with different glycol concentrations in aqueous solution. *P*_0_ is the initial pressure of gas phase in the sapphire cell, the mass fraction of ZIF-8 in slurry was specified to 0.2, and temperature equaled to 293.15 K.

When using the mixing solvent, the permeability of membrane should vary with the change of solvent compositions. Hence, we can further tune the osmotic pressure by using the mixed solvents of water and glycol. As shown by Supplementary Fig. S3, for CH_4_, there is no remarkable decrease of the sorption ability of the ZIF-8 slurry when adding less than 20 wt% glycol to water. However, when the content of glycol reaches 40 wt%, we observed remarkable increase of osmotic pressure. As shown by Supplementary Fig. S4, the sorption ability of ZIF-8/water-glycol slurry for C_2_H_6_ or C_2_H_4_ keeps unchanged until the concentration of glycol is higher than 60 wt%. On the other side, the sorption rate always decreases with the increase of glycol concentration (Fig. 4). For the capture of C_2_H_6_ or C_2_H_4_, 20 wt% or so might be an ideal concentration at which the sorption rate and adsorption capacity of C_2_H_6_ or C_2_H_4_ keep as high as those for pure water case.

**Figure 5:**
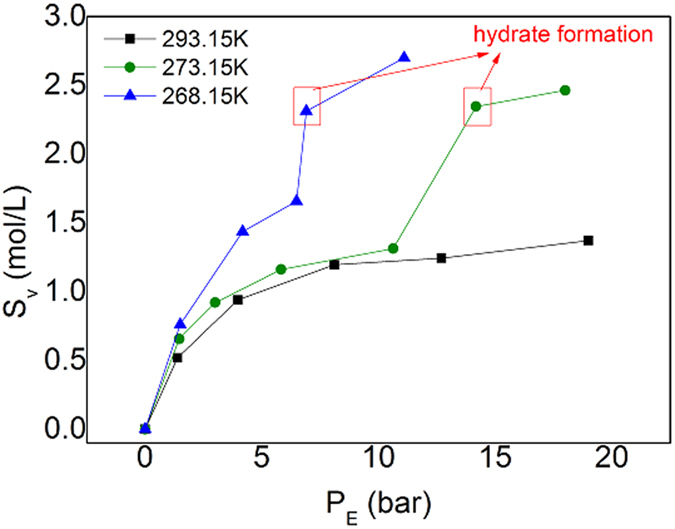
Comparison of ab(d)sorption isotherms of C_2_H_4_ on ZIF-8/water-glycol slurry at different temperatures. Both the mass fraction of ZIF-8 in slurry and glycol in aqueous solution were specified to 0.2.

Addition of suitable quantity of glycol to water has several other advantages. At first, the presence of glycol can inhibit the agglomeration among ZIF-8 particles and prevent them from precipitating from slurry. As shown by Supplementary Fig. S5a, ZIF-8 particles in pure water are easy to agglomerate and deposit on the wall of the equilibrium cell while little precipitation was observed when using aqueous glycol solution (Supplementary Fig. S5b). Supplementary Figure S6 shows the ZIF-8 particle size distribution in the aqueous solution of 20 wt% glycol. We found most particles’ sizes are around 500 nm, implying that the slurry is very fine. Additionally, the ZIF-8/water slurry foams seriously during its desorption while this phenomenon declined in the presence of glycol (Supplementary Fig. S5c and d). Foaming is unfavorable for the application of slurry in the industry. The final advantage using water-glycol mixing solvent is the slurry can be used below the ice point. It is very interesting to find that the sorption capacity of ZIF-8/water-glycol slurry for C_2_H_6_ or C_2_H_4_ increases dramatically below the ice point, as shown in Fig. 5. One can see that, from 293.15 K to 273.15 K, there is only slight increase of the sorption ability of slurry for C_2_H_4_. However, the further decrease of temperature of only 5 K, dramatic increase of the sorption capacity was observed. This result demonstrates that we can get high C_2_H_4_ capture efficiency only a little below the ice point. This kind of increase of the sorption capacity might be caused by the capillary condensation of C_2_H_4_ in the pores of ZIF-8 at lower temperatures. It should be noted that the rapid increase of the sorption capacity after the circled point shown in Fig. 5 was caused by the formation of C_2_H_4_ clathrate hydrate. Although formation of hydrate could increase the sorption ability of slurry, it also increases the viscosity of the system. Hence, it should be controlled deliberately in practical application.

### Enhancing Separation of Gas Mixture by Tuning ZIF-8 Slurry

Separation of low boiling gas mixtures, e.g. natural gas (CH_4_/C_2_H_6_), coal bed gas (CH_4_/N_2_), pyrolysis gas (CH_4_/C_2_H_4_/C_2_H_6_/C_3_H_6_/H_2_) involved in the production of C_2_H_4_, tail gas of synthesis ammonia apparatus (H_2_/N_2_/CH_4_/Ar), and refinery dry gases (CH_4_/C_2_H_4_/C_2_H_6_/N_2_/H_2_/CO_2_) is widely involved in energy and petrochemical industries. As stated above, most gas species involved in these gas mixtures have certain osmotic pressures on ZIF-8/glycol slurry except for C_3_H_6_. For CO_2_, we can eliminate the osmotic pressure by adding methylpyrazole (mIm) to glycol and it could then be separated from other gas species with high efficiency^42^. Solubilities of C_2_H_4_ or C_2_H_6_ in both ZIF-8/glycol slurry and ZIF-8/glycol-mIm slurry are very low unless their partial pressures are high enough. This means they cannot be removed from gas mixture sufficiently using these slurries unless the operation pressure is very high. C_3_H_6_ might be separated from gas mixtures containing lighter components (H_2_, N_2_, CH_4_, C_2_H_4_, and C_2_H_6_) by using ZIF-8/glycol slurry sufficiently. However, it can also be separated from them by using ZIF-8/water slurry or ZIF-8/water-glycol slurry efficiently. As two later slurries have lower viscosities (Supplementary Fig. S7) and higher sorption rates, they should be the better choices in practical use with respect to the separation of this kind of gas mixtures. Thus we focus our work on the application of ZIF-8/water slurry and ZIF-8/water-glycol slurry.

**Figure 6:**
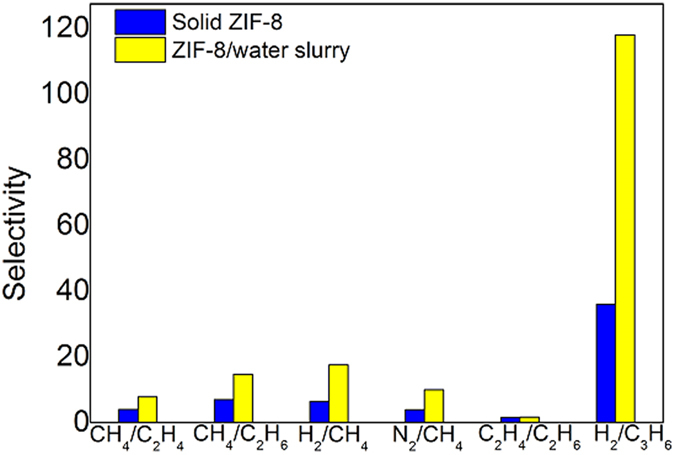
Comparison of the separation selectivity of solid ZIF-8 and ZIF-8/water slurry for CH_4_/C_2_H_4_, CH_4_/C_2_H_6_, H_2_/CH_4_, N_2_/CH_4_, C_2_H_4_/C_2_H_6_, and H_2_/C_3_H_6_ binary gas mixtures.

We first performed a group of experiments on the separation of several typical binary gas mixtures, CH_4_/C_2_H_4_, CH_4_/C_2_H_6_, H_2_/CH_4_, N_2_/CH_4_, C_2_H_4_/C_2_H_6_, and H_2_/C_3_H_6_, by using solid ZIF-8 and ZIF-8/water slurry respectively. The results are summarized in Fig. 6 and more detailed experimental results are given in Supplementary Table S2. We can see that, because of the membrane effect, the separation factors by using ZIF-8/water slurry are always higher than those by using solid ZIF-8. For H_2_/C_3_H_6_, the separation factor reaches 118, which is more than three times higher than that by using solid ZIF-8. The concentration of C_3_H_6_ in gas phase can be decreased from 36.44 mol% to 2.95 mol% through one equilibrium stage of separation, indicating a very high capture efficiency of C_3_H_6_. The separation of H_2_/C_3_H_6_ might be involved in C_3_H_6_ dehydrogenation process. For CH_4_/C_2_H_4_, CH_4_/C_2_H_6_, H_2_/CH_4_, and N_2_/CH_4_, the separation factors are also doubled compared with the cases using solid ZIF-8. They reached 7.81, 14.7, 17.7, and 9.96 respectively. Separations of CH_4_/C_2_H_6_, H_2_/CH_4_, and N_2_/CH_4_ are directly involved in the purifications of natural gas, recycling H_2_ produced in hydrocracking process of oil, and coal bed gas, respectively. Thus these results are of practical significance.

In order to show the critical role of liquid membrane on the high separation selectivity of ZIF-8 slurries, we calculated the contributions of absorption and adsorption to the separation factor with respect to the separation of the H2/CH4 mixture. Here we assume that gas components in three phases (gas, liquid and solid) are in simple thermodynamic equilibrium without the presence of liquid membrane and the liquid absorbent and solid adsorbent in the slurry do not affect each other. In this case, the selectivity of the slurry is the weighted average of the selectivity of the (pure) liquid absorbent and that of (pure) solid adsorbent^46^. The calculated results based on the above assumptions are compared with the experimental ones in Supplementary Fig. S8. One can see that the actual selectivity of the slurry are much higher than those of both pure solid ZIF-8 phase and pure water phase as well as the weighted average value of them. Obviously, the increment of the selectivity of the slurry should be attributed to the role of liquid membranes surrounding ZIF-8 particles. Similar phenomenon has been found in our previous work^42^.

As C_2_H_4_ is very important raw material for the production of large number of chemicals, the low cost separation of C_2_H_4_ is strongly concerned by petrochemical industries. In most cases, C_2_H_4_ is accompanied by C_2_H_6_ in gas mixture, e.g., pyrolysis gas and catalytic cracking (FCC) dry gas. As C_2_H_6_ is the best raw material for producing C_2_H_4_, its capture is also very important. As an example of practical use of ZIF-8 slurry, here we put emphasis on the capture of C2 (C_2_H_4_ + C_2_H_6_) from catalytic cracking dry gas CH_4_/C_2_H_6_/C_2_H_4_/N_2_/H_2_. As pyrolysis gas CH_4_/C_2_H_6_/C_2_H_4_/N_2_/H_2_ is the sub system of FCC dry gas, the results obtained here are also applicable for separation of pryolysis gas.

**Figure 7:**
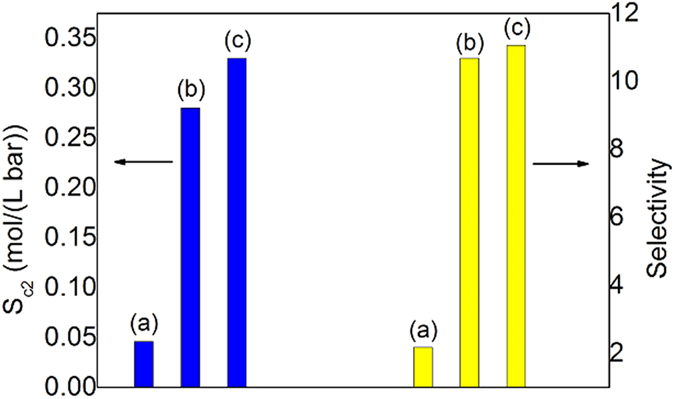
Separation performance of different media for FCC dry gas. Comparison of the separation abilities of (**a**) ZIF-8/glycol, (**b**) ZIF-8/water, and (**c**) ZIF-8/water-glycol slurries with respect to feed gas CH_4_/C_2_H_6_/C_2_H_4_/N_2_/H_2_ (24.83/7.24/19.82/26.71/21.40 mol%) mixture at 293.15 K, where both the mass fraction of ZIF-8 in slurry and glycol in aqueous solution were specified to 0.2.

The feed gas CH_4_/C_2_H_6_/C_2_H_4_/N_2_/H_2_ (24.83/7.24/19.82/26.71/21.40 mol%) was synthesized according to the typical composition of catalytic cracking dry gas. At first, we compared the C2 capture efficiency of different slurry materials, ZIF-8/glycol, ZIF-8/water, ZIF-8/water-glycol with a glycol mass fraction of 0.2 in aqueous phase. The detailed experimental results were shown in Supplementary Table S3 and summarized in Fig. 7. One can see that ZIF-8/water-glycol has the highest sorption capacity (*S*_C2_) and the highest capture selectivity (*S*) of C2 over other components. As expected, the ZIF-8/glycol slurry has very low sorption capacity because of higher osmotic pressures, i.e. the sorption impetus is too low for gas molecules to enter the pores of ZIF-8.

As shown by Supplementary Table S4, the solubility coefficient of C2 (*SC*_*2*_) on ZIF-8/water slurry decreases with the increase of its partial pressure. For practical application, the higher gas/absorbent ratio is always critical for decreasing the separation cost. The gas/absorbent ratio is determined by the solubility coefficient of object gas species. Thus how to increase *S*_C2_ is very important. Figure 5 shows that there is a dramatic increase of the sorption capacity of ZIF-8/water-glycol slurry below the ice point. We therefore performed separation experiments at 269.15 K and higher pressure of 2.98 MPa using ZIF-8/water-glycol slurry. As shown by Supplementary Table S4, *S*_C2_ increased more than three times when temperature decreased from 293.15 K to 269.15 K under 29 bar or so. The separation selectivity also increased obviously. However, Supplementary Table S5 shows that *S*_C2_ increases little when temperature changes from 303.15 K to 283.15 K. This is consistent with Fig. 5. The dramatic increase of the sorption capacity below the ice point not only implies that we can use a much larger gas-slurry flux ratio, e.g., 140 V/V, but also indicates we can use temperature swing sorption process besides of PSA.

**Figure 8:**
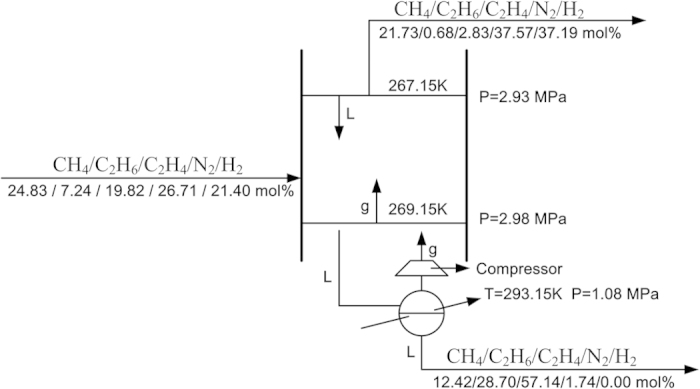
A simulation of three-stage separation of FCC dry gases by using ZIF-8/water-glycol slurry. The mass fraction of ZIF-8 in slurry and glycol in aqueous solution were specified to 0.2.

For evaluating multistage separation efficiency of ZIF-8/water-glycol slurry, we performed separation experiments simulating a three-stage separation involving both temperature swing and pressure swing with respect to the capture of C2 from FCC dry gas. To do so, another two feed gas samples CH_4_/C_2_H_6_/C_2_H_4_/N_2_/H_2_ (25.06/2.99/8.03/34.19/29.78 mol%) and CH_4_/C_2_H_6_/C_2_H_4_/N_2_/H_2_ (25.12/17.89/42.51/12.99/1.49 mol%) were synthesized according to the gas compositions of gas phase and slurry phase produced in the separation of feed gas CH_4_/C_2_H_6_/C_2_H_4_/N_2_/H_2_ (24.83/7.24/19.82/26.71/21.40 mol%) at 269.15 K and 2.98 MPa. The former one was separated under same pressure of 2.98 MPa and a little lower temperature of 267.15 K. The later one was separated under lower pressure of 1.08 MPa and higher temperature of 293.15 K. In fact, this separation represent a flash of slurry produced in the initial separation of feed gas CH_4_/C_2_H_6_/C_2_H_4_/N_2_/H_2_ (24.83/7.24/19.82/26.71/21.40 mol%) under 2.98 MPa. The experimental results for three equilibrium stage separation were shown in Supplementary Table S6. The whole three-stage separation was summarized in Fig. 8. We can see that through a three-stage separation, C2 can be enriched from 27.06 to 86.05 mol% in slurry phase, while the content of C2 in gas phase could be reduced to only 3.7 mol%, indicating most C2 can be captured. It is reasonable enough to anticipate a more sufficient capture and a higher purity of C2 if using a multistage (>3) separation in a traditional absorption tower. As shown in Supplementary Fig. S7, the viscosity of ZIF-8/water-glycol slurry is lower than that of pure liquid glycol. ZIF-8/water-glycol slurry is really liquid-like. Additionally, as stated above, both sorption rate and desorption rate of gas on ZIF-8/water-glycol slurry is very high. Thus, it will be easy to realize multistage separations like distillation in widely used absorption towers.

### Regeneration of Slurry

Essential to any gas capture material is the energy required for gas release. Here the regeneration properties of both ZIF-8/water and ZIF-8/water-glycol slurry were also investigated. The separation ability of these two kinds of slurries that regenerated under atmosphere pressure and that regenerated by vacuuming at room temperature were shown in Supplementary Tables S7 and S8. For ZIF-8/water slurry, ~65% of its separation ability could be regenerated at atmosphere pressure and the full capacity can be regained by vacuuming at just 293.15 K. For ZIF-8/water-glycol slurry, its full capacity also could be easily regained by vacuuming at just 293.15 K. We found the sorption ability of slurry did not change after several times of cycling use. As a matter of fact, most experiments were performed using the regenerated ZIF-8 slurry (if we do not need to change the solvent) or regenerated ZIF-8 by vacuum vaporing (if we need to change the solvent). The X-ray diffraction patterns and scanning electron microscopy images of the regenerated ZIF-8 also show the structure of ZIF-8 is perfectly retained (Supplementary Fig. S9 and S10). From Fig. S9 we can see that the sizes of most ZIF-8 particles are around 300 nm, which are smaller than the average diameter of ZIF-8 particles in the slurry, i.e. 500 nm or so, as shown by Supplementary Fig. S6. In fact the particle sizes shown in Fig. S6 are apparent values. The increment of the apparent particle size when ZIF-8 is suspended in glycol aqueous solution proves the existence of liquid membrane surrounding the ZIF-8 particles in certain extent.

## Conclusions

Several important insights emerge in this work. When porous material like ZIF-8 suspended in suitable solvents whose molecules are too large to enter the pores of solid particles, solvent molecules self-assemble liquid membrane surrounding solid particles. Compared with traditional solid membrane, the self-assembly liquid membrane will never be destroyed and its permeability is fully tunable by adjusting the composition of solvent. The strength of the membrane is determined by the interaction between the solvent molecules and the function group on the surface of solid particles. Just like traditional solid membrane, there also exist obvious and different osmotic pressures between two sides of liquid membrane for different gas molecules. This is attributed to the large difference between the concentration of gas molecules in solvent phase and solid phase, which results in a reverse gas concentration gradient across the liquid membrane, i.e., the gas concentration at the inner boundary might be larger than that at the outer boundary of the membrane. Hence a positive excess pressure, i.e., osmotic pressure, is required for the transfer of gas molecules through the liquid membrane from outside to inside. The strength or permeability of liquid membrane for specific gas species could be tuned aiming at increasing the apparent separation selectivity of the slurry formed by porous material and solvent. Surrounding ZIF-8 particles, glycol molecules form tighter membrane, indicated by higher osmotic pressures for most gas species lighter than propene. Water molecules form looser film because of the hydrophobic behavior of ZIF-8 and osmotic pressures emerge only for very light gas species like N_2_ and H_2_. When using the mixed solvent composed of water and glycol, the osmotic pressure changes with the increase of glycol concentration. By this way, the apparent separation selectivity of ZIF-8 slurry becomes tunable. When tuning the separation selectivity of ZIF-8 slurry, other factors should also be taken into account. In positive aspect, besides increasing separation selectivity, adding glycol to water makes the slurry more stable and finer. It can also inhibit the emergence of serious foaming during desorption. In addition, it can decrease the freezing point of slurry and allow the slurry to work below the ice point (273.15 K) where dramatic increase of sorption capacity may occur. In negative aspect, adding glycol to water results in an increase of viscosity and a decrease of sorption/desorption rate. One should balance the positive factors and negative ones when determining the content of glycol.

When using ZIF-8/solvent slurry as absorbent for gas mixture separation, three single separation processes, i.e. absorption, membrane, and adsorption, are integrated into one pseudo-absorption process. Due to the membrane effect of liquid film, enhanced separation could be achieved compared with simple absorption or simple adsorption. Our separation experiments on different kinds of gas mixtures show that ZIF-8/water slurry always manifests remarkable higher separation selectivity than solid ZIF-8 and it can be tuned to further enhance the capture of light hydrocarbons by adding suitable quantity of glycol to water. ZIF-8/water-glycol slurry is really liquid-like as diameters of most ZIF-8 particles are 500 nm or so. When the glycol concentration in liquid is controlled to be below than 20 wt%, its viscosity is obviously lower than that of pure liquid glycol. More importantly, it has high sorption/desorption rate. We believe it can be readily used to separate different kinds of low boiling gas mixtures under or near normal temperature, by applying traditional absorption towers with multiple gas-liquid equilibrating stages. Especially, we found for light hydrocarbons (C_2_H_4_, C_2_H_6_, etc.) at temperature only 4 K below the ice point, ZIF-8/water-glycol slurry has high sorption capacity and selectivity, indicating capture of light hydrocarbons might become cheaper if using ZIF-8/water-glycol slurry compared with traditional separation approaches like PSA, middle cooling oil absorption, cryogenic separation, etc. We think ZIF-8/water-glycol slurry approach may revolutionize the separation of low boiling gas mixture because it takes the advantages of distillation, membrane, and adsorption together.

## Methods

### Materials

Materials used in this work include ZIF-8, glycol, water, and feed gases. Among them, ZIF-8 was purchased from Sigma-Aldrich. Glycol was purchased from Beijing Chemical Reagents Company, China. Analytical grade nitrogen (99.99%), methane (99.99%), ethylene (99.99%), ethane (99.99%), propylene (99.99%) and hydrogen (99.999%) were purchased from Beijing AP Beifen Gas Industry Company, China. The feed gas mixtures CH_4_/C_2_H_4_, CH_4_/C_2_H_6_, H_2_/CH_4_, N_2_/CH_4_, C_2_H_4_/C_2_H_6_, H_2_/C_3_H_6_, and CH_4_/C_2_H_6_/C_2_H_4_/N_2_/H_2_ were prepared by ourselves. A Hewlett-Packard gas chromatograph (HP 7890) was used to analyze the composition of the prepared gas mixtures.

### Ab(d)sorption measurements

All the ab(d)sorption measurement experiments were performed using the experimental apparatus as schematically illustrated in Supplementary Fig. S11. A detailed description of the setup can be found in our previous report^42^. The key parts of the apparatus are a transparent sapphire cell and a steel-made blind cell, which are both installed in an air bath. The effective volume of the sapphire cell is 60 cm^3^ and that of the blind cell plus tubes connecting to it is 112 cm^3^. The maximum working pressures of these two cells are designed to be 20 MPa. To directly observe samples in the cell, a luminescence source of type LG100H is mounted on the outside of the cell. A secondary platinum resistance thermometer (type-pt100) is used as the temperature sensor. A calibrated Heise pressure gauge and differential pressure transducers are used to measure the system pressure. The uncertainties of pressure and temperature measurements are ±0.01 MPa and ±0.1 K, respectively. Real-time readings of the system temperature and pressure are recorded by a computer.

Before the experiments, the sapphire cell was dismounted from the apparatus, washed with distilled water and dried, then loaded with a known quantity of dry porous material. After that, a known amount of solvent was immersed into the sapphire cell slowly and evenly. Both the used dry porous material and solvent were weighed by an electrical balance with a precision of ±0.1 mg. The mixture of porous material and liquid solvent was stirred to form a suspension mixture (i.e., slurry). Subsequently, the cell was installed back onto the apparatus. The system (sapphire cell + blind cell + tubes connecting two cells) was then purged through vacuuming. Enough amount of feed gas was injected into the blind cell, then the desired value of temperature was set through the air-bath. Once both temperature and pressure of the blind cell were kept constant, the pressure of gas mixture in the blind cell was recorded as the initial pressure 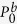. The top valve of the sapphire cell was opened slowly then, letting the feed gas flow into the sapphire cell from the blind cell until the pressure in the sapphire cell reached the desired value, which was recorded as 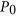. Afterwards, the top valve was closed and the magnetic stirrer was turned on. The pressure of the residual gas in the blind cell was recorded as 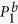. With the sorption of the slurry, the pressure in the sapphire cell decreased gradually. During each measurement, the variation of pressure in the sapphire cell with the elapsed time was recorded. When the system pressure remained constant for at least 2 hours, we considered the equilibrium of system was achieved. The equilibrium pressure of the sapphire cell was recorded as *P*_E_. Gas mixture in the equilibrium gas phase of the sapphire cell was sampled under constant pressure by pushing the connected hand pump and analyzed by a HP 7890 gas chromatograph. The volume of the slurry in the sapphire cell can be obtained by measuring the height of the equilibrium liquid phase. The inner radius of the sapphire cell is known to be 1.27 cm. In this work, the sorption amount of one gas species in the slurry was determined through mass balance as described below.

The total mole number (*n*_t_) of the feed gas mixture that was injected into the sapphire cell is calculated by the following formula:


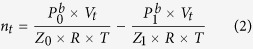


where *T* is the system temperature, 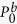 is the initial pressure in the blind cell, 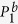 is the residual pressure in the blind cell after injecting the gas into the sapphire cell, *V*_t_ is the total volume of the blind cell plus tubes connecting to it, and *R* is the universal gas constant. Compressibility factors *Z*_0_ and *Z*_1_ were calculated using the Benedict-Webb-Rubin-Starling equation of state. The total gas amount (*n*_E_) in the gas phase of the sapphire cell after sorption equilibrium is determined by:


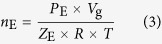


where *P*_E_ is the equilibrium pressure of the sapphire cell and *Z*_E_ is the compressibility factor corresponding to *T* , *P*_E_, and equilibrium gas composition. *V*_g_ is the volume of equilibrium gas phase in the sapphire cell. The total sorption amount of one gas species *i* in the slurry is calculated as,


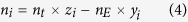


where *z*_i_ and *y*_i_ are the mole fractions of gas species *i* in the feed gas and equilibrium gas phase, respectively. Its apparent mole fraction in the equilibrium slurry phase then can be obtained by the following formula:


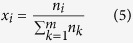


where *m* is the component number of gas phase. The net adsorption amount (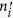) of one gas species in solid ZIF-8 suspended in solvent is calculated as,


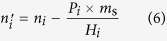


where *H*_i_ is Henry constant of one gas species in solvent, *P*_i_ is the partial pressure of one gas species in gas phase, and *m*_s_ is the mass of solvent in the slurry. Henry constants of CH_4_, C_2_H_6_, C_2_H_4_, C_3_H_6_, N_2_, and H_2_ in water and those in glycol at 293.15 K were experimentally determined and listed in Supplementary Table S1.

The net adsorption capacity of ZIF-8 in the slurry for a gas species i is calculated as,


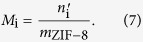


where *m*_ZIF-8_ is the mass of ZIF-8 in the slurry.

The initial vapor-liquid volume ratio is defined as


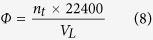


where *V*_L_ is the volume of ZIF-8/liquid slurry. For binary feed gas mixtures, apparent selectivity (*S*) of the slurry is calculated with,


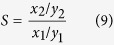


In this work the apparent volume solubility coefficient of C2 (C_2_H_6_ + C_2_H_4_), *S*_c2_, in the slurry and the apparent selectivity (*S*) of C2 over all other gas components (CH_4_ + N_2_ + H_2_) together are used to characterize the ability of the slurry recovering C2 from multi-component feed gas mixtures, CH_4_ (1)/C_2_H_6_ (2)/C_2_H_4_ (3)/N_2_ (4)/H_2_ (5),


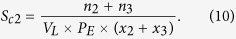



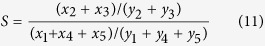


### Characterization

The adsorbents (solid ZIF-8) were characterized by XRD (SIMADU XRD 6000) with Cu Kα radiation (0.1542 nm, 40 kV and 400 mA) at a scanning rate of 2 °C per minute. The morphologies and energy dispersive X-ray spectroscopy measurements were obtained using a FEI Quanta 200F scanning electron microscope.

### Physical properties determination

The size distribution of samples in ZIF-8/liquid slurry was measured by virtue of the Zetasizer Nano-ZS laser nanoparticle-size analyzer manufactured by the Britain Malvern Instruments. The viscosity of ZIF-8 slurry were measured by using a modified Cannon-Ubbelohde suspended level capillary viscometers of (0.8 mm) in diameter^47^.

## Additional Information

**How to cite this article**: Liu, H. *et al*. Tunable integration of absorption-membrane-adsorption for efficiently separating low boiling gas mixtures near normal temperature. *Sci. Rep*. **6**, 21114; doi: 10.1038/srep21114 (2016).

## Supplementary Material

Supplementary Information
