# Supplementary material for: Tunable integration of absorption-membrane-adsorption for efficiently separating low boiling gas mixtures near normal temperature
Source: Sci Rep. 2016 Feb 19;6:21114. doi: 10.1038/srep21114 (PMC4759557; doi:10.1038/srep21114)
Supplement: Supplementary Information [file srep21114-s1.pdf]

Supplementary Information:
Tunable integration of absorption-membrane-adsorption for efficiently separating low boiling gas mixtures near normal temperature

Huang Liu[1,2], Yong Pan[1], Bei Liu[1*], Changyu Sun[1], Ping Guo[2], Xueteng Gao[1], Lanying Yang[1], Qinglan Ma[1] & Guangjin Chen[1*]

1. State Key Laboratory of Heavy Oil Processing, China University of Petroleum, Beijing 102249, P. R. China

2. State Key Laboratory of Oil and Gas Reservoir Geology and Exploitation, Southwest Petroleum University, Chengdu 610500, China

*Corresponding Authors: liub@cup.edu.cn (B. Liu), gjchen@cup.edu.cn (G. Chen).

Supplementary figures

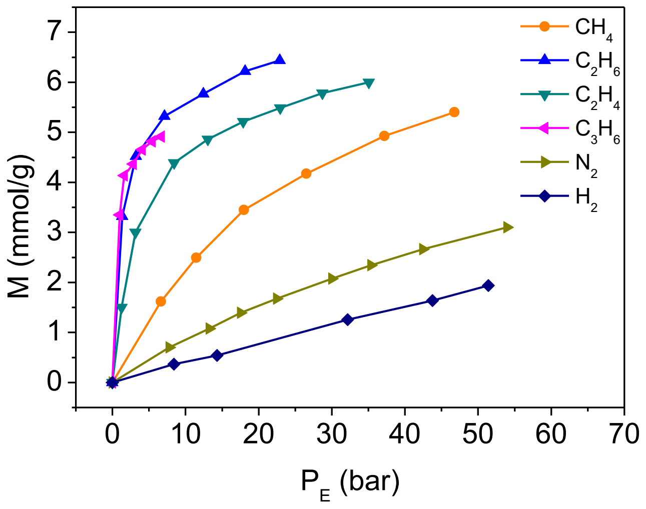

Figure S1: Adsorption isotherms of different gas species CH_4, C_2H_6, C_2H_4, C_3H_6, N_2, and H_2 on solid ZIF-8 at 293.15 K.

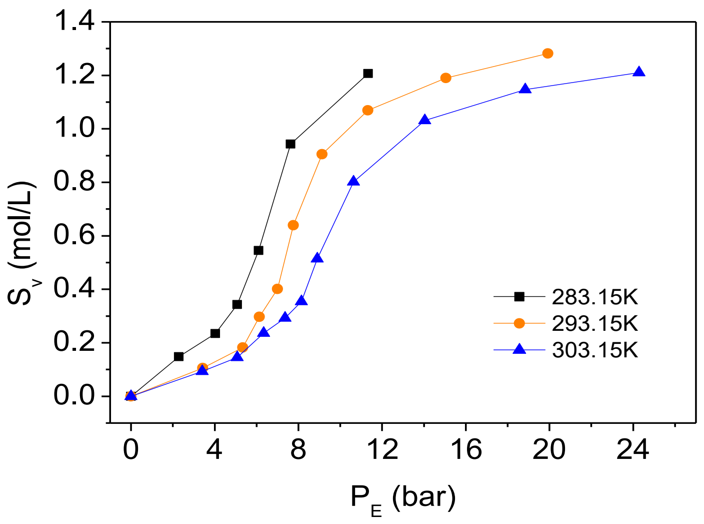

(a)

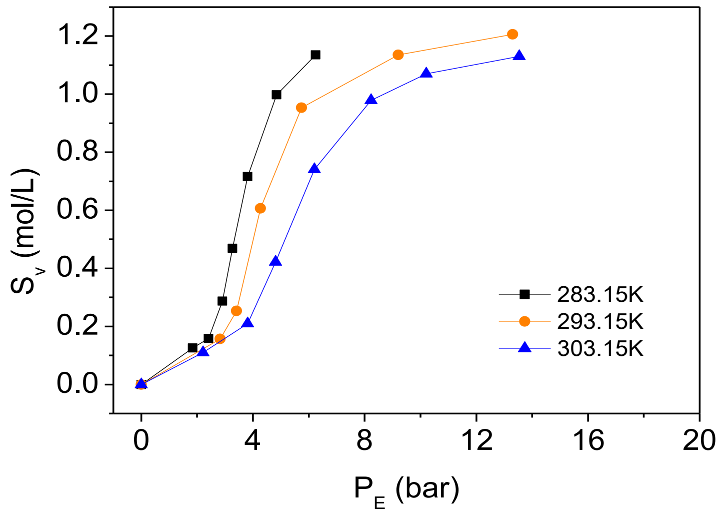

(b)

Figure S2: Influence of temperature upon the shape of sorption isotherms of (a) ethylene and (b) ethane in ZIF-8/glycol slurry.

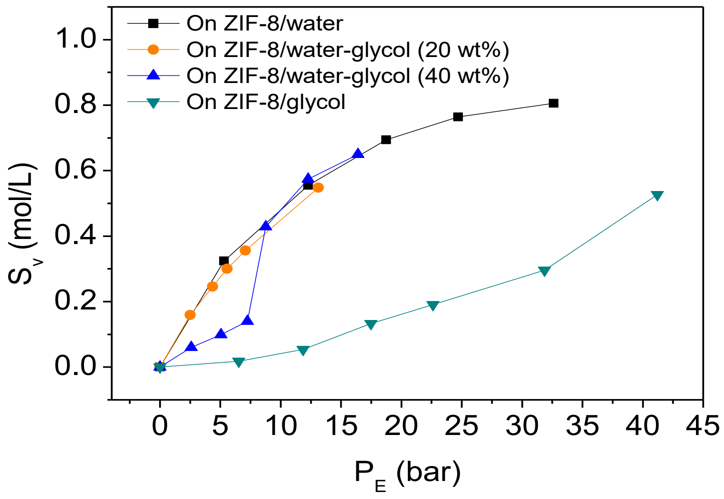

Figure S3: Comparison of the solubility of CH_4 in ZIF-8/water, ZIF-8/water-glycol with the mass fraction of glycol in aqueous solution equaled to 0.2 and 0.4, and in ZIF-8/glycol slurries at 293.15 K, where the mass fraction of ZIF-8 in slurry was specified to 0.2.

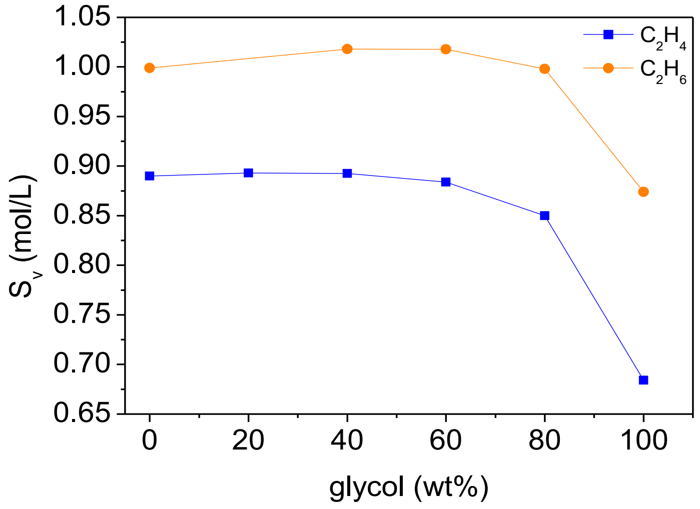

Figure S4: The solubility of ethane and ethylene in ZIF-8/water-glycol slurry with different glycol content in aqueous solution (wt) at 293.15 K with nearly the same initial impetus (~ 12.5 bar) in the reactor, where the mass fraction of ZIF-8 in slurry was specified to 0.2

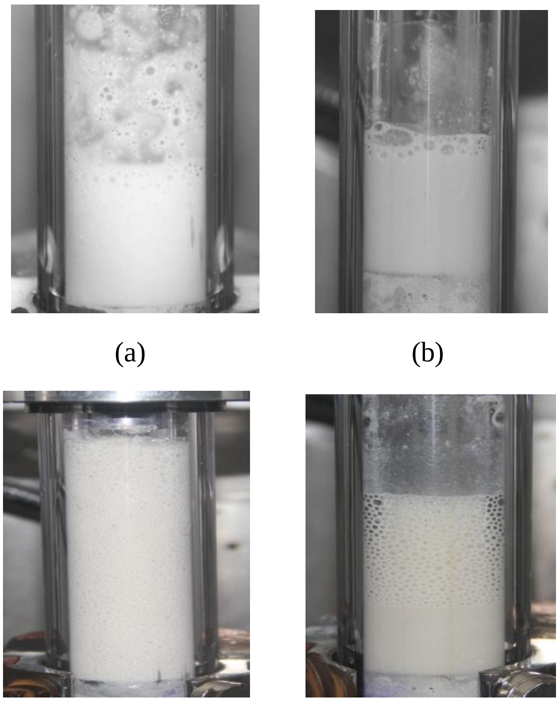

(a) (b) (c) (d)

Figure S5: The morphology of ZIF-8/liquid slurry. (a) Fresh ZIF-8/water slurry. (b) Fresh ZIF-8/water-glycol slurry. (c) Gas desorption process for ZIF-8/water slurry. (d) Gas desorption process for ZIF-8/water-glycol slurry. The mass fraction of glycol in aqueous solution was specified to 0.2.

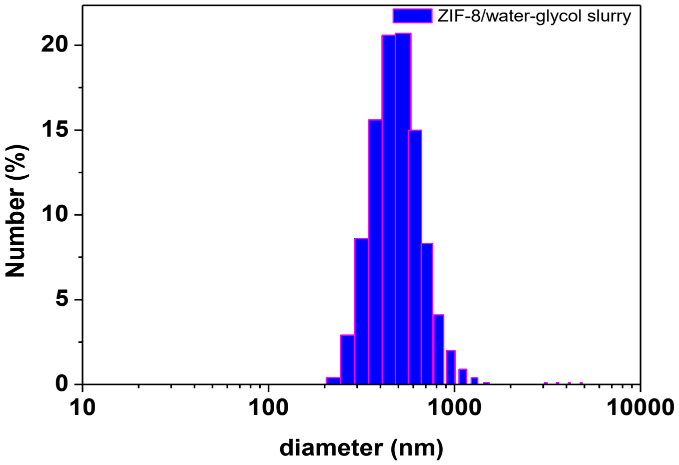

Figure S6: The particle diameter distributions of ZIF-8/water-glycol slurry, where the mass fraction of glycol in aqueous solution was specified to 0.2.

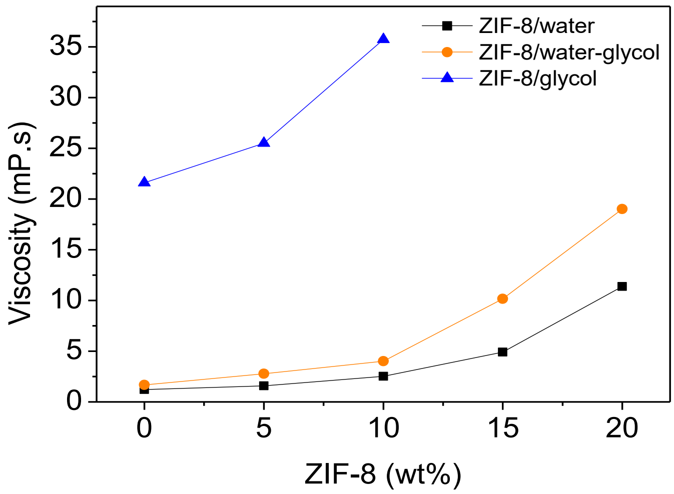

Figure S7: The viscosity of ZIF-8/water, ZIF-8/water-glycol, and ZIF-8/glycol slurries at 293.15 K with different mass fractions of ZIF-8 (wt%). The mass fraction of glycol in water-glycol solution was specified to 0.2.

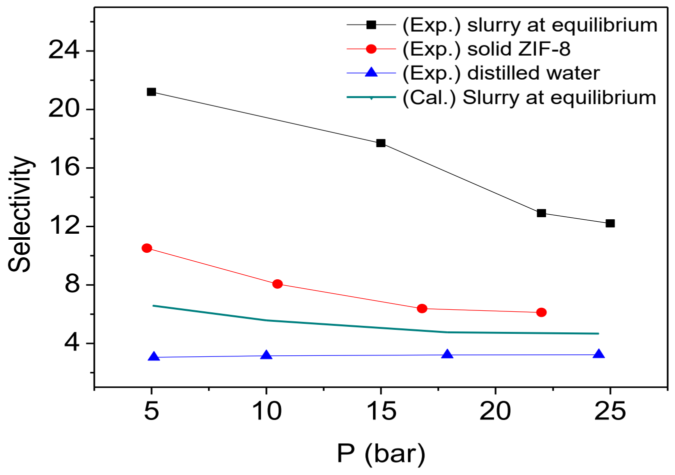

Figure S8: The experimental measured H_2/CH_4 (65.72/34.28 mol%) selectivity at 293.15 K in ZIF-8/water slurry, solid ZIF-8 and distilled water. For comparison, we have calculated the corresponding weighted selectivity in ZIF-8/water slurry.

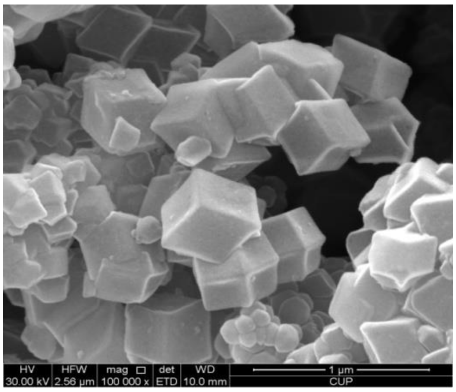

(a)

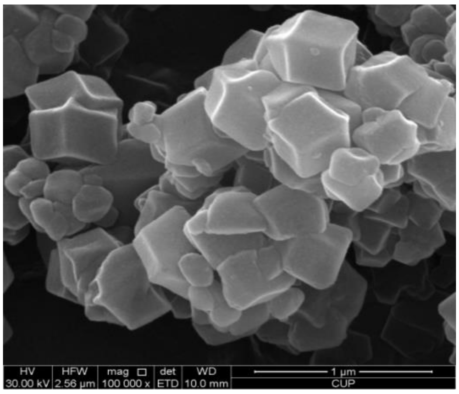

(b)

Figure S9: Comparison of the SEM images of (a) fresh solid ZIF-8, (b) recovered ZIF-8 from ZIF-8/water slurry after being used for three times in separating $CH_4/C_2H_6/C_2H_4/N_2/H_2$ (24.83/7.24/19.82/26.71/21.40 mol%) gas mixture at 293.15 K.

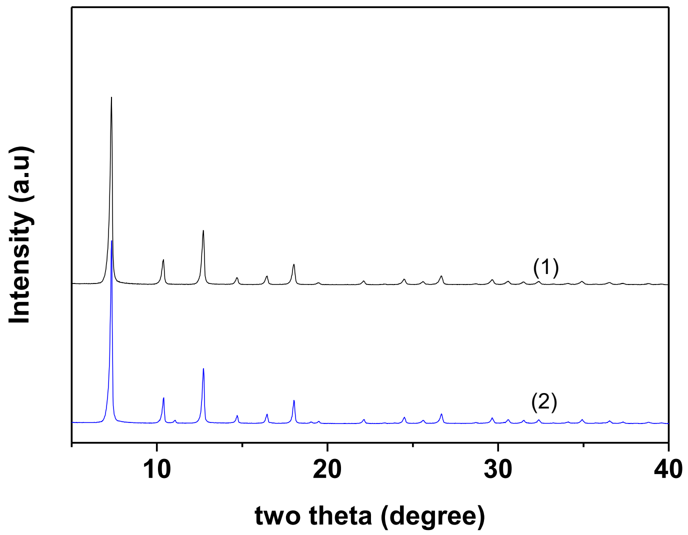

Figure S10: Comparison of the experimental XRD patterns of (1) fresh solid ZIF-8 between, (2) recovered ZIF-8 from ZIF-8/water slurry after being used for three times in separating $CH_4/C_2H_6/C_2H_4/N_2/H_2$ (24.83/7.24/19.82/26.71/21.40 mol%) gas mixture at 293.15 K.

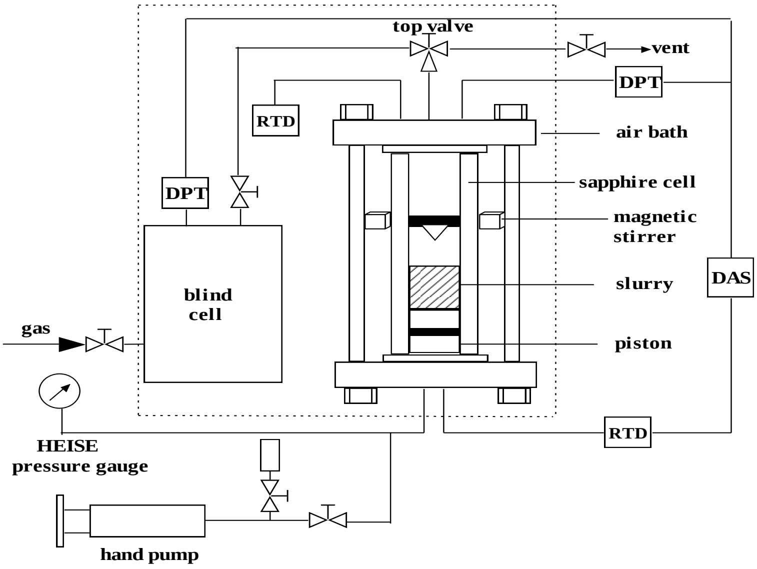

Figure S11: Schematic diagram of the experimental apparatus. RTD, resistance thermocouple detector; DPT, differential pressure transducer; and DAS, data acquisition system.

Supplementary Tables

Table S1: Henry constant (*H*) of gas components in water (H_1) and glycol (H_2) at 293.15 K.

Gas components	H_1 (bar g mmol^{-1})	H_2 (bar g mmol^{-1})
CH_4	703	147
C_2H_6	489	60.0
C_2H_4	190	49.8
C_3H_6	152	18.6
N_2	1488	482
H_2	1263	377

Table S2: Comparison of the separation results for CH_4/C_2H_4, CH_4/C_2H_6, H_2/CH_4, N_2/CH_4, C_2H_4/C_2H_6, and H_2/C_3H_6 mixtures by using solid ZIF-8 and ZIF-8/water slurry at 293.15 K.

mediums[a]	P_0 (bar)	P_E (bar)	y_2 (mol%)	x_2 (mol%)	S
For CH_4/C_2H_4 (25.22/74.78) mixture					
Solid ZIF-8	7.41	4.39	65.57	88.36	3.99
ZIF-8/water slurry	7.07	3.67	59.46	91.97	7.81
For CH_4/C_2H_6 (34.78/65.22) mixture					
Solid ZIF-8	8.03	4.39	47.66	86.38	6.96
ZIF-8/water slurry	6.64	3.23	38.74	90.30	14.7
For H_2/CH_4 (65.72/34.28 mol%) mixture					
Solid ZIF-8	19.3	16.8	28.10	71.34	6.37
ZIF-8/water slurry	18.1	15.4	25.20	85.54	17.7
For N_2/CH_4 (55.81/44.19 mol%) mixture					
Solid ZIF-8	14.6	12.3	39.44	71.66	3.92
ZIF-8/water slurry	12.3	10.0	35.52	84.59	9.96
For C_2H_4/C_2H_6 (27.95/72.05 mol%) mixture					
Solid ZIF-8	10.2	5.42	67.71	76.71	1.57
ZIF-8/water slurry	7.74	3.29	65.89	76.44	1.68
For H_2/C_3H_6 mixture[b]					
Solid ZIF-8	4.41	2.51	2.96	51.98	35.5
ZIF-8/water slurry	3.62	2.00	2.95	78.22	118

[a] The same amount of ZIF-8 was used in both single adsorption and absorption-adsorption hybrid separation processes. The mass faction of ZIF-8 in ZIF-8/water slurry was specified to 0.2.

[b] When using solid ZIF-8, the mole fraction of C_3H_6 in feed gas equaled to 33.21 mol%, and when using ZIF-8/water slurry, the mole fraction of C_3H_6 in feed gas equaled to 36.44 mol%.

Table S3: Separation results for $CH_4/C_2H_6/C_2H_4/N_2/H_2$ (24.83/7.24/19.82/26.71/21.40 mol%) mixture on ZIF-8/glycol, ZIF-8/water, and ZIF-8/water-glycol slurry at 293.15 K. Both the mass fraction of ZIF-8 in slurry and glycol in aqueous solution were all specified to 0.2.

Mediums	P_0	P_E	Equilibrium gas phase, mol%					Slurry phase, mol%					S	S_{c2}
	(bar)	(bar)	y_1	y_2	y_3	y_4	y_5	x_1	x_2	x_3	x_4	x_5		(mol L^{-1} bar^{-1})
ZIF-8/glycol	12.5	10.7	22.45	6.57	17.72	30.05	23.21	36.91	10.70	30.51	9.67	12.21	2.18	0.05
ZIF-8/water	12.5	8.91	24.31	2.84	10.30	33.43	29.12	26.15	18.45	43.53	9.41	2.46	10.8	0.28
ZIF-8/water-glycol	12.2	8.29	22.51	2.54	9.48	34.51	30.96	29.45	17.62	42.55	8.59	1.79	11.1	0.33

Table S4: Separation results for $CH_4/C_2H_6/C_2H_4/N_2/H_2$ (24.83/7.24/19.82/26.71/21.40 mol%) mixture on ZIF-8/water slurry under different pressures, where the mass faction of ZIF-8 in slurry was specified to 0.2. Temperature is 293.15 K except for the noted one.

Φ	P_E	Equilibrium gas phase, mol%					Slurry phase, mol%					S	S_{c2}
	(bar)	y_1	y_2	y_3	y_4	y_5	x_1	x_2	x_3	x_4	x_5		(mol L^{-1} bar^{-1})
22	4.82	23.74	2.65	9.81	33.62	30.18	27.46	17.52	42.25	10.70	2.07	10.4	0.43
38	8.91	24.31	2.84	10.30	33.43	29.12	26.06	18.39	43.39	9.58	2.58	10.7	0.28
68	16.5	25.46	3.54	12.08	31.70	27.22	22.86	18.62	43.59	11.40	3.53	8.90	0.19
101	26.4	26.60	4.11	13.29	30.79	25.21	17.14	20.79	48.01	9.08	4.98	10.5	0.14
149[a]	29.8	24.61	2.86	8.44	32.72	31.37	24.76	16.68	44.31	12.91	1.34	12.3	0.41

[a] Temperature for this experiment run was set to 269.15 K.

Table S5: Separation results for $CH_4/C_2H_6/C_2H_4/N_2/H_2$ (24.83/7.24/19.82/26.71/21.40 mol%) mixture on ZIF-8/water-glycol slurry at four different temperatures. Both the mass fraction of ZIF-8 in slurry and glycol in aqueous solution were all specified to 0.2.

T	P_0	Φ	P_E	Equilibrium gas phase, mol%					Slurry phase, mol%					S	S_{c2}
(K)	(bar)		(bar)	y_1	y_2	y_3	y_4	y_5	x_1	x_2	x_3	x_4	x_5		(mol L^{-1} bar^{-1})
303.15	12.1	36	8.58	22.67	2.85	10.15	34.74	29.59	30.34	18.26	43.48	6.03	1.89	10.8	0.27
293.15	12.2	37	8.29	22.51	2.54	9.47	34.52	30.96	29.45	17.62	42.55	8.59	1.79	11.1	0.33
283.15	11.9	37	8.02	22.40	2.56	9.85	34.31	30.88	29.24	17.15	40.82	9.91	2.88	9.74	0.34

Table S6: Separation results for three $CH_4/C_2H_6/C_2H_4/N_2/H_2$ mixtures on ZIF-8/water-glycol slurry to simulate a three-stage separation process. Both the mass fraction of ZIF-8 in slurry and glycol in aqueous solution were all specified to 0.2.

T	P_0	Φ	P_E	Equilibrium gas phase, mol%					Slurry phase, mol%					S	S_{c2}
(K)	(bar)		(bar)	y_1	y_2	y_3	y_4	y_5	x_1	x_2	x_3	x_4	x_5		(mol L^{-1} bar^{-1})
For $CH_4/C_2H_6/C_2H_4/N_2/H_2$ (24.83/7.24/19.82/26.71/21.40 mol%)															
269.15	41.0	149	29.8	24.61	2.86	8.44	32.73	31.36	24.75	16.68	44.29	12.94	1.34	12.3	0.41
For $CH_4/C_2H_6/C_2H_4/N_2/H_2$ (25.06/2.99/8.03/34.16/29.76 mol%)															
267.15	36.8	110	29.3	21.73	0.68	2.83	37.57	37.19	36.35	10.85	25.71	22.62	4.47	15.8	0.43
For $CH_4/C_2H_6/C_2H_4/N_2/H_2$ (25.12/17.89/42.51/12.99/1.49 mol%)															
293.15	17.8	57	10.8	33.71	10.52	32.50	20.62	2.65	12.42	28.70	57.14	1.74	0.00	8.03	0.21

Table S7: Test the regeneration ability of ZIF-8/water slurry for the separation of $CH_4/C_2H_6/C_2H_4/N_2/H_2$ (24.83/7.24/19.82/26.71/21.40 mol%) mixture at 293.15 K.

P_0 (bar)	Φ	P_E (bar)	Equilibrium gas phase, mol% y_1	y_2	y_3	y_4	y_5	Slurry phase, mol% x_1	x_2	x_3	x_4	x_5	S	S_{c2} (mol L^{-1} bar^{-1})
Fresh ZIF-8/water slurry														
12.5	38	8.91	24.31	2.84	10.30	33.43	29.12	26.06	18.39	43.39	9.58	2.58	10.7	0.28
By using slurry that recovered under atmosphere pressure and room temperature														
12.3	37	9.17	23.93	3.95	12.61	32.13	27.38	27.51	17.15	41.51	10.41	3.42	7.15	0.18
By using slurry that recovered through vacuuming at room temperature														
12.1	37	8.48	24.34	2.98	10.46	33.18	29.04	26.03	17.91	43.19	10.55	2.32	10.1	0.27

Table S8: Test the regeneration ability of ZIF-8/water-glycol slurry for the separation of $CH_4/C_2H_6/C_2H_4/N_2/H_2$ (24.83/7.24/19.82/26.71/21.40 mol%) mixture at 293.15 K.

P_0 (bar)	Φ	P_E (bar)	Equilibrium gas phase, mol%					Slurry phase, mol%					S	S_{c2} (mol L^{-1} bar^{-1})
			y_1	y_2	y_3	y_4	y_5	x_1	x_2	x_3	x_4	x_5		
Fresh ZIF-8/water-glycol slurry														
12.2	37	8.29	22.51	2.54	9.47	34.52	30.96	29.45	17.62	42.55	8.59	1.79	11.1	0.33
By using slurry that recovered once through vacuuming at room temperature														
12.1	37	8.27	22.63	2.55	9.82	34.21	30.79	29.15	17.51	42.09	9.44	1.81	10.5	0.32
By using slurry that recovered twice through vacuuming at room temperature														
12.3	37	8.42	22.56	2.56	9.41	34.75	30.72	29.75	17.95	43.43	7.34	1.53	11.7	0.33
